# Crosstalk Between the Gut Microbiota and Epithelial Cells Under Physiological and Infectious Conditions

**DOI:** 10.3389/fcimb.2022.832672

**Published:** 2022-01-27

**Authors:** An Zhou, Yi Yuan, Min Yang, Yujiao Huang, Xin Li, Shengpeng Li, Shiming Yang, Bo Tang

**Affiliations:** ^1^ Department of Gastroenterology, Xinqiao Hospital, Third Military Medical University, Chongqing, China; ^2^ Institution of Basic Medicine, Third Military Medical University, Chongqing, China; ^3^ The First Clinical College, ChongQing Medical University, Chongqing, China

**Keywords:** gut microbiota, epithelial cells, infection, COVID-19, probiotics

## Abstract

The gastrointestinal tract (GIT) is considered the largest immunological organ, with a diverse gut microbiota, that contributes to combatting pathogens and maintaining human health. Under physiological conditions, the crosstalk between gut microbiota and intestinal epithelial cells (IECs) plays a crucial role in GIT homeostasis. Gut microbiota and derived metabolites can compromise gut barrier integrity by activating some signaling pathways in IECs. Conversely, IECs can separate the gut microbiota from the host immune cells to avoid an excessive immune response and regulate the composition of the gut microbiota by providing an alternative energy source and releasing some molecules, such as hormones and mucus. Infections by various pathogens, such as bacteria, viruses, and parasites, can disturb the diversity of the gut microbiota and influence the structure and metabolism of IECs. However, the interaction between gut microbiota and IECs during infection is still not clear. In this review, we will focus on the existing evidence to elucidate the crosstalk between gut microbiota and IECs during infection and discuss some potential therapeutic methods, including probiotics, fecal microbiota transplantation (FMT), and dietary fiber. Understanding the role of crosstalk during infection may help us to establish novel strategies for prevention and treatment in patients with infectious diseases, such as *C. difficile* infection, HIV, and COVID-19.

## Introduction

The GIT presents an enormous area to the exterior environment, and the epithelium of the GIT forms physical and biochemical barriers between multicellular animals and the exterior environment, which protect the mucosa and peripheral organs from pathogenic microorganisms and toxins. On the other hand, a tremendously diverse gut microbiota inhabits the GIT, which plays an essential role in human health and disease (Fan and Pedersen, 2021). Some probiotics, such as *Lactobacillus rhamnosus* GG (LGG), can enhance intestinal epithelial functions by increasing the expression of ZO-1 and occludin proteins (Han et al., 2019). IECs segregate the host immune system and the gut microbiota to avoid unnecessary immune responses and intestinal inflammation, thereby maintaining the homeostasis of the GIT (Soderholm and Pedicord, 2019). However, some exogenous pathogens may alter the components of the microbiota, destroy the epithelial barrier, and induce some infectious diseases in GIT. For instance, *Clostridium difficile* infection (CDI) is recognized as a major cause of antibiotic-associated diarrhea, and the risk factors for CDI include antibiotics, proton pump inhibitors and age (Samarkos et al., 2018). These risk factors induce the dysbiosis of the gut microbiota, promote the colonization of *C. difficile* on colonic epithelium, and lead to apoptosis and necrosis of epithelial cells. Recent studies have suggested that cigarette smoke can disturb the lung microbiota, reduce epithelial integrity, and increase susceptibility to respiratory pathogens (Heijink et al., 2012; Greathouse et al., 2018). Although this evidence has indicated alterations in the microbiota and epithelial cells during infection in the GIT, the crosstalk between the microbiota and epithelial cells has not been clarified thus far. In this review, we will focus on the possible crosstalk between epithelial cells and the microbiota under physiological and infectious conditions and some potential therapeutic strategies targeting the microbiota, which may provide a new approach for the prevention and treatment of infectious diseases of the GIT.

## Crosstalk Between the Gut Microbiota and IECs Under Physiological Conditions

**Figure 1:**
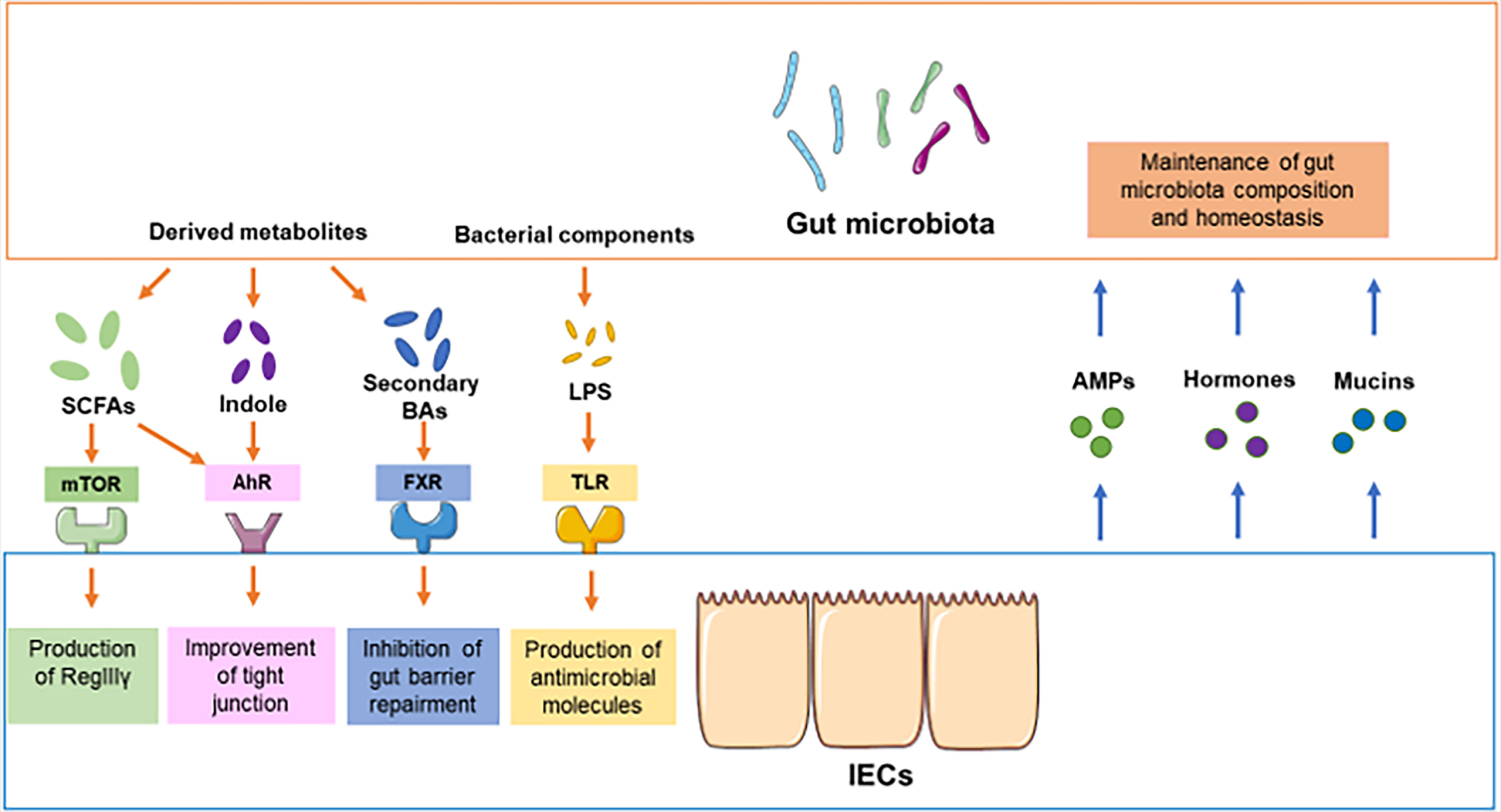
Crosstalk between the gut microbiota and IECs under physiological conditions. In physiological conditions, the components of the gut microbiota and derived metabolites compromise or destroy intestinal barrier integrity through different pathways, such as mTOR, AhR, FXR and TLR. For instance, SCFAs promote the production of RegIIIγ and defensins from IECs by activating mTOR. The activation of SCFAs and indole can improve the function of tight junctions. The components of the gut microbiota, such as LPS, promote the production of antimicrobial molecules in IECs. On the other hand, secondary bile acids from gut microbiota may inhibit the repair of the gut barrier. Conversely, IECs can modulate the composition of gut microbiota by providing some signaling molecules or alternative energy resources, including AMPs, hormones, and mucins.

In humans, the GIT represents a large microbial ecosystem, housing approximately 10^11^ gut microbiota (Sender et al., 2016). The gut microbiota plays a critical role in intestinal endocrine functions, maturation of immune cells, and protection against pathogen overgrowth (Lynch and Pedersen, 2016). IECs, including Paneth cells, absorptive epithelial cells, and goblet cells, construct two types of mucosal barriers that segregate immune cells and gut microbiota to maintain homeostasis and prevent inflammation in the GIT. In addition, gut microbiota can increase the epithelial defense mechanism and reduce intestinal permeability to form a mucosal barrier (Berg et al., 2015). Interactions between the gut microbiota and IECs are crucial for the maintenance of intestinal homeostasis. Some studies suggest that interactions between the gut microbiota and IECs are key regulators of epithelial permeability by modulating the tight junctions (Allam-Ndoul et al., 2020). Homeostasis in the GIT can also be orchestrated by the circadian clock and signals transduced by the gut microbiota (Mukherji et al., 2013). In the following section, we will discuss the signals from the gut microbiota to IECs and the regulation of the gut microbiota by IECs (**Figure 1**).

### Signals From the Gut Microbiota to IECs

The gut microbiota can produce hundreds of metabolites and proteins that modulate some functions in humans, including immune system development, nutrient processing, and maintenance of energy homoeostasis (Blaak et al., 2020). Some bacterial metabolites have been identified as playing important roles in the regulation of IECs and maintenance of the gut epithelial barrier. For instance, short-chain fatty acids (SCFAs), a group of metabolites derived from the bacterial fermentation of dietary fibers, are crucial for the gut barrier by regulating mucus production and the luminal pH and providing fuel for epithelial cells (Martin-Gallausiaux et al., 2021). In addition, SCFAs can modulate the differentiation and proliferation of IECs to strengthen gut barrier functions and as host metabolism. SCFAs include propionic acid, butyric acid, and acetic acid. An increasing number of studies have indicated the essential role of SCFAs in the maintenance of the intestinal epithelial barrier. For example, Zhao et al. suggested that oral feeding with SCFAs could promote the production of RegIIIγ and defensins from IECs by activating mammalian target of rapamycin (mTOR) and signal transducer and activator of transcription 3 (STAT3) in IECs in mice, which provides a novel pathway by which SCFAs regulate IEC expression and intestinal homeostasis (Zhao et al., 2018). In addition, Bilotta et al. found that propionate could promote IEC migration, epithelial renewal, and repair by enhancing cell speed and persistence in mice (Bilotta et al., 2021). Furthermore, aryl hydrocarbon receptor (AhR) is regarded as an essential regulator of immune processes in the GIT, but the role of AhR activation in IECs has only started to be understood. Emerging evidence highlights that activation of AhR in IECs enhances gut barrier functions by enhancing tight junctions through the regulation of Notch1 and increasing the expression of the IL10 receptor, which protects mice from colitis (Liu et al., 2018; Yu et al., 2018). A recent work previously demonstrated that butyrate could enhance the expression of AhR-related genes through histone deacetylase inhibitor (HDACi) activities (Jin et al., 2017), and Ludovica et al. identified the novel role of butyrate as an AhR ligand in human IECs, which elucidated the potential mechanism of IEC regulation by SCFAs (Marinelli et al., 2019). In addition, hexokinase (HK), which catalyzes the phosphorylation of glucose, is a key enzyme in the first step of glycolysis. HK2 is highly expressed in IECs, contributes to immune responses, and is upregulated during inflammation. Recent studies have suggested that microbiota-derived butyrate represses HK2 expression through histone deacetylase 8 (HDAC8) and reduces mitochondrial respiration, which provides a therapeutic avenue for GIT inflammation (Hinrichsen et al., 2021). Collectively, SCFAs not only provide fuel for epithelial cells but also enhance the gut barrier by activating some signaling pathways, such as mTOR, STAT3 and AhR.

On the other hand, emerging evidence suggests that tryptophan-derived gut microbiota metabolites play a critical role in maintaining the balance of the gut barrier. The gut microbiota can convert dietary tryptophan into indole by tryptophanase, and indole can be recognized by AhR in IECs (Hubbard et al., 2015). Li et al. evaluated the effect of indole-3-propionic acid (IPA) on the gut barrier in a Caco-2/HT29 coculture model (Li et al., 2021). These authors demonstrated that IPA strengthens the gut barrier by increasing goblet cell secretion products (RELMβ, TFF3) and mucins (MUC2 and MUC4). In addition, a recent study indicated that three metabolites derived from the gut microbiota, indole-3-ethanol, indole-3-pyruvate, and indole-3-aldehyde, could protect the gut barrier by maintaining the junctional complex and associated regulatory proteins (ezrin, myosin IIA) and that the effects are mediated by AhR (Scott et al., 2020). Furthermore, Swimm et al. indicated that administration of indole-3-carboxaldehyde (ICA) protects and repairs the gut barrier from damage by reducing transepithelial bacterial translocation *via* type I interferon (IFN1) signaling in graft-versus-host disease (GVHD) (Swimm et al., 2018). It was demonstrated that indole could also upregulate the expression of cell junction-associated molecules (claudins, occludin) by activating the pregnane X receptor (PXR) (Venkatesh et al., 2014). This evidence indicates that activation of AhR, IFN1 and PXR in IECs by indole enhances gut barrier integrity.

In addition, primary bile acids, such as chenodeoxycholic acid (CDCA) and cholic acid (CA), play a crucial role in lipid digestion, cholesterol metabolism and regulatory pathways in the host (Chiang, 2009). Primary bile acids are synthesized from cholesterol in the liver through a complex process involving more than 14 enzymes (Chiang, 2009). Approximately 95% of bile acids are reabsorbed by IECs, and unabsorbed bile acids can serve as substrates for gut microbial metabolism and transform to secondary bile acids, such as deoxycholic acid (DCA), lithocholic acid and ursodeoxycholic acid (UDCA) (Hofmann, 1999). However, secondary bile acids can be harmful to the GIT and induce some intestinal diseases, such as inflammatory bowel disease (IBD) and neonatal necrotizing enteritis (Halpern et al., 2006). Recent studies demonstrated that DCA could inhibit IEC proliferation by activating the farnesoid X receptor (FXR) and inhibit wound healing of the gut barrier (Mroz et al., 2018). In addition, secondary bile acids can activate extracellular signal-regulated kinase 1 and 2 (ERK1/2) signaling and β-catenin signaling *via* the c-myelocytomatosis (c-Myc) and activator protein 1 (AP1) target pathways, which stimulate the proliferation and invasiveness of colon cancer cells (Pai et al., 2004). In contrast, some studies have suggested that UDCA is beneficial to the repair of the epithelial barrier *via* the epidermal growth factor receptor (EGFR) pathway (Golden et al., 2018). DCA can stimulate the G protein–coupled receptor Takeda G protein receptor 5 (TGR5) in the gut epithelial layer to promote colonic peristalsis by releasing calcitonin gene-related peptide (CGRP) and 5-hydroxytryptamine (5-HT) (Alemi et al., 2013). Therefore, secondary bile acids derived from the gut microbiota can influence the proliferation of IECs and colonic peristalsis through different pathways, including FXR, ERK1/2, EGFR, and TGR5.

In addition to the metabolites of the gut microbiota, some bacterial components, such as flagellin and lipopolysaccharide (LPS), contribute to the proliferation and production of cytokines and antimicrobial molecules in IECs *via* NOD-like receptors (NLRs) and Toll-like receptors (TLRs) (Kayama et al., 2020). Furthermore, some gut microbiota can attach to the mucosal surface, which is considered bacterial adhesion, and induce specific gene expression in IECs. For instance, segmented filamentous bacteria (SFB) can attach to IECs and induce the production of serum amyloid A (SAA) by IECs, which stimulates the differentiation of T helper 17 (Th17) cells and contributes to resistance against bacterial infection (Liang et al., 2006; Ivanov et al., 2009). On the other hand, gut microbiota can also regulate lipid absorption and export in IECs by modulating the circadian transcription factor NFIL3 through the STAT3 pathway (Wang et al., 2017). This study established an essential link among the gut microbiota, the IECs’ circadian clock, and metabolism of the host. Taken together, these findings indicate that metabolites of gut microbiota, bacterial components and bacterial adhesion play a crucial role in the maintenance of the gut barrier integrity, proliferation of IECs and protection against bacterial infection in the GIT.

### Regulation of Gut Microbiota by IECs

Previous evidence has identified the role of gut microbiota in the regulation of IECs by different signals, and inversely, IECs can also regulate and maintain the homeostasis of the gut microbiota. The IEC monolayer is composed of different cell types, including absorptive enterocytes, goblet cells, Paneth cells and enteroendocrine cells (von Moltke et al., 2016). IECs form a barrier between the gut microbiota and the rest of the body, and different subtypes of IECs modulate the gut microbiota by releasing type 2 immune mediators, mucins, antimicrobial peptides (AMPs) and hormones (Gehart and Clevers, 2019). For instance, Paneth cells can produce AMPs and regenerate the islet-derived 3 (Reg3) family of proteins to segregate gut microbiota and IECs and avoid unnecessary immune responses (Ayabe et al., 2000; Vaishnava et al., 2011). Serotonin production by enterochromaffin cells has been reported to modulate the composition of gut microbiota (Kwon et al., 2019). In addition, David et al. indicated that increasing the expression of indoleamine 2,3-dioxygenase 1 (IDO1) in IECs promotes the differentiation of secretory sells and alters the composition of gut microbiota by modulating the AhR and Notch signaling pathways, which elucidated the important role of IDO1 in GIT homeostasis (Alvarado et al., 2019). Furthermore, a recent work reported a novel role of IEC-derived liver kinase B1 (LKB1) in suppressing colitogenic microbiota by modulating the expression of IL-18, which decreased the susceptibility to dextran sodium sulfate (DSS)-induced colitis in a mouse model (Liu et al., 2018). Forkhead box protein O1 (Foxo1), a key regulator in the modulation of mucus secretion by goblet cells, plays an essential role in the maintenance of the intestinal barrier. However, loss of Foxo1 in IECs results in defects in goblet cell mucus secretion and autophagy, inducing dysbiosis of the gut microbiota and the GIT microenvironment (Chen et al., 2021). This study demonstrated the crucial role of Foxo1 in the establishment and maintenance of gut barrier integrity by regulating goblet cell function. In addition, autophagy is an important catabolic recycling pathway that degrades proteins and organelles *via* lysosomes to maintain the homeostasis of cells. Although impaired autophagy is closely correlated with Crohn’s disease (CD), the role of autophagy in the regulation of gut microbiota is poorly understood. Yang et al. found that impaired autophagy of IECs dramatically reduced alpha diversity and altered the composition of gut microbiota in an autophagy-related 5 (Atg5)-knockout mouse model (Yang et al., 2018).

On the other hand, diet‐derived components, such as polysaccharides and glycans, are the main nutrient source for gut microbiota (Flint et al., 2012). However, IECs can also provide an alternative energy source for some gut microbiota when dietary glycans are exhausted, including glycans on mucin proteins and the surface of IECs. Especially in the distal intestine, where dietary glycans derived from food are insufficient, only resident gut microbiota that adhere to the glycans on the mucus layer can develop as a rich microbial habitat (Li et al., 2015). For instance, several *Clostridiales* members use mucin‐associated sialic acid and the sugar fucose as energy sources, promoting their colonization of the GIT (Wlodarska et al., 2017). Symbiotic *B. fragilis* strains markedly upregulate glycan-degrading genes and obtain more nutrients than other gut microbiota in the colonic lumen (Donaldson et al., 2020). In addition, *A. muciniphila* is dependent on mucus nutrients, and the change in metabolic pathways of *A. muciniphila* occurs when mucin is poorly expressed, which improves anti-obesity activities in mice (Shin et al., 2019). An increasing number of studies have demonstrated that gut microbiota can influence mucin glycosylation and mucosal homeostasis *via* glycosidases, metabolites, and bacterial components. In contrast, mucin glycans also influence the behavior of gut microbiota, including probiotic traits, energy acquisition and signal transmission (Qu et al., 2021). Taken together, this evidence elucidates the essential role of IECs in modulation of the gut microbiota and maintenance of homeostasis of the GIT through different signaling pathways, AMPs, and mucins.

In summary, the interactions between gut microbiota and IECs are complicated. Although these studies mentioned above have demonstrated signals from the gut microbiota to IECs or regulation of the gut microbiota by IECs under physiological conditions, more evidence is required to elucidate the underlying mechanism of the crosstalk between gut microbiota and IECs. In the next section, we will focus on the interactions during infectious conditions and elucidate the underlying mechanisms in depth.

## The Interactions Between Gut Microbiota and IECs that Protect from Infection

**Figure 2:**
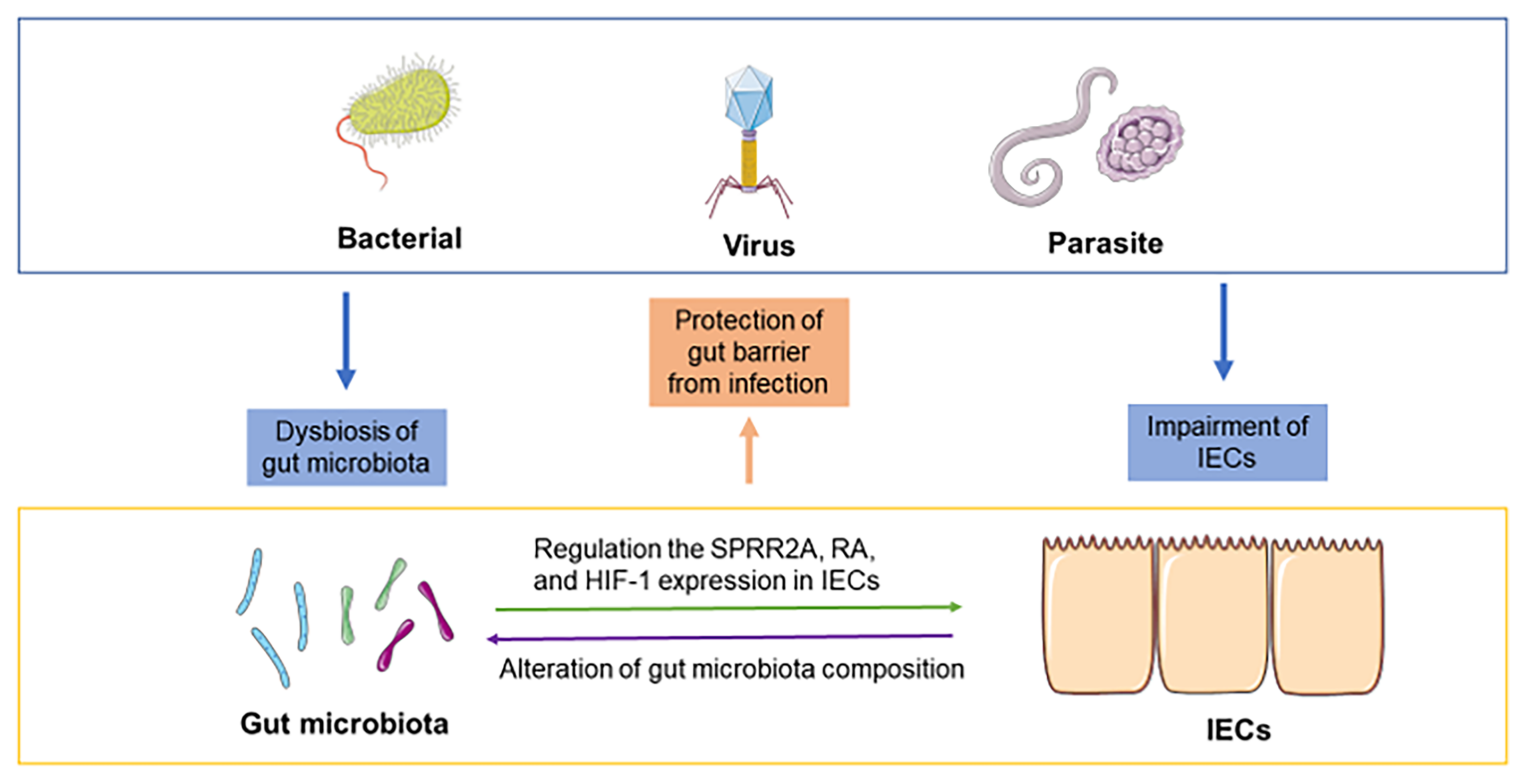
Crosstalk between the gut microbiota and IECs during infection. Bacterial infection, viral infection and parasite infection can induce dysbiosis of the gut microbiota and impairment of the gut barrier. The crosstalk between the gut microbiota and IECs may play an essential role during infection. For instance, the gut microbiota regulates the response to pathogens in IECs by modulating the expression of SPRR2A, RA, and HIF-1. IECs can alter the composition of gut microbiota and contribute to protection against infections.

Enteric infections have profound effects on intestinal nutrition, absorption, and childhood development (Petri et al., 2008). Infections are the most common complications in patients with hemorrhagic shock, serious burns and major surgery (Wang et al., 2019). In addition, enteric infections (bacteria, viruses or parasites) can alter the composition of gut microbiota, destroy tight junctions and increase the permeability of the gut barrier (Wang et al., 2019). In contrast, the gut microbiota and IECs are critical in resisting colonization by pathogenic microorganisms. Although the mechanisms by which the gut microbiota provides colonization resistance (CR) are still unknown, some evidence suggests that the gut microbiota can inhibit the colonization of exogenous microorganisms through nutrient competition, bacteriophage deployment, secretion of antimicrobial products, and support of gut barrier integrity (Ducarmon et al., 2019). IECs can maintain physical barrier function and release some antimicrobial molecules, mucus, and carbohydrate moieties into the lumen, which prevent the aberrant attachment of pathogens (Goto and Ivanov, 2013). Furthermore, the crosstalk between gut microbiota and IECs during infection may also play an essential role in preventing infection by pathogens (**Figure 2**).

### Signals From the Gut Microbiota to IECs

Infections by exogenous microorganisms can alter the microenvironment of the GIT, including disturbances of the gut microbiota and IECs. In that case, the gut microbiota transmits some associated signals to IECs and contributes to defense against infection. For instance, a recent study indicated that small proline-rich protein 2A (SPRR2A), a novel intestinal antibacterial protein, could protect against helminth-induced bacterial invasion of gut barriers (Hu et al., 2021). SPRR2A is induced by gut microbiota and secreted in Paneth cells and goblet cells, which selectively kill gram-positive bacteria by disrupting their membranes (Hu et al., 2021). In addition, segmented filamentous bacteria (SFB) are commensal gut microbiota that protect against some pathogens, such as *C. rodentium* (Chung et al., 2012). Unlike the majority of the gut microbiota that are spatially separated from IECs, SFB can bind to IECs in the distal small intestine (Atarashi et al., 2015). Although previous studies have demonstrated that SFB could decrease *C. rodentium* infection through the expansion of CD4+ Th17 cells in mice (Goto et al., 2014), Vivienne indicated that SFB colonization induced epigenetic modifications of IECs at retinoic acid receptor (RAR) motifs and enhanced defense through retinoic acid (RA) signals, which provided a new approach to prevent and combat infections (Woo et al., 2021). Mayara et al. suggested that the gut microbiota belonging to Clostridia could regulate RA concentration in the GIT by suppressing the expression of retinol dehydrogenase 7 (Rdh7) in IECs and preventing colonization of *Salmonella typhimurium* (Grizotte-Lake et al., 2018). Furthermore, the gut microbiota shields the host against infections by providing CR. Alteration of the gut microbiota and various drugs, such as antidiabetics, proton pump inhibitors, and antibiotics, can induce the disruption of CR and ultimately cause infection (Ducarmon et al., 2019). A recent work suggested that prior infections lead to an increase in the resistance of gut microbiota to subsequent infection (Stacy et al., 2021). The study found that infections induce taurine production and that gut microbiota can convert taurine to sulfide and ultimately inhibit pathogen respiration, which contributes to the maintenance of CR. Mahesh et al. demonstrated that the gut microbiota resorts to mucus glycoproteins as a nutrient source and disrupts the colonic mucus barrier during dietary fiber deficiency, which promotes greater epithelial access to exogenous microorganisms, such as *C. rodentium* (Desai et al., 2016). This research elucidated the relationship among dietary fiber, gut barrier dysfunction and the gut microbiota and indicated the benefits of dietary therapeutics to protect against infection. In addition to dietary fiber, a recent work suggested that treatment with hyaluronan alleviated *C. rodentium*-induced bacterial colitis by modulating the diversity of the gut microbiota and preventing the colonization of *C. rodentium* in IECs, which provides novel insight into the important role of hyaluronan in enteric infection through the interaction between gut microbiota and IECs (Mao et al., 2021). On the other hand, metabolites from the gut microbiota, such as SCFAs, also play a crucial role in combatting enteric pathogen infection. For example, SCFAs can increase O2 consumption by IECs and contribute to intestinal hypoxia, which is important for barrier function and metabolism of IECs (Pral et al., 2021). Previous studies have reported that butyrate could regulate the expression of hypoxia-inducible factor-1 (HIF-1) in IECs and promote the production of tight junction proteins and that HIF-1 signaling provided protection in *C. difficile*-induced colitis (Hirota et al., 2010; Yin et al., 2020). Recent evidence has demonstrated that interventions with butyrate relieve *C. difficile*-induced colitis through the HIF-1 pathway (Fachi et al., 2019). An acetate-yielding diet promoted changes in the gut microbiota during infection to healthy conditions and contributed to epithelial repair *via* the immune program (Yap et al., 2021). This evidence elucidated the mechanisms by which SCFAs modulate the crosstalk between the gut microbiota and IECs during infection.

In addition to bacterial infections (*C. difficile, C. rodentium* and *Salmonella typhimurium*), understanding the potential mechanisms of interaction between the gut microbiota and IECs during viral infection is also crucial to provide novel therapeutic strategies for virus infection, especially for the COVID-19 pandemic. Accumulating evidence has demonstrated that both antagonistic and promoting effects are found in the gut microbiota during viral infection. The gut microbiota can bind viruses, wash them out from IECs, and modulate the production of immune molecules to suppress the viruses (Lei et al., 2016). In contrast, viruses can bind to some bacterial products and increase virion stability (Li et al., 2015). In addition, virus infection can alter the composition and diversity of the gut microbiota. For instance, Ren et al. found alterations in the oral microbiota and gut microbiota in patients with COVID-19, which contributed to establishing a diagnostic model for COVID-19 (Ren et al., 2021). Yun et al. indicated that the gut microbiota is involved in the magnitude of COVID-19 severity by modulating the host immune responses (Yeoh et al., 2021). However, the mechanisms by which SARS-CoV-2 influences the crosstalk between the gut microbiota and IECs are still unclear, and some hypotheses have suggested that COVID-19 could disturb the homeostasis of the gut microbiota, increase the release of proinflammatory cytokines and destroy the tight junctions of IECs (Penninger et al., 2021). In addition, a recent study suggested that rotavirus infection induced an increase in *Bacteroides* and *Akkermansia*, which digest mucin and decrease the number of filled goblet cells in the small intestine (Engevik et al., 2020). This finding points to a potential role of gut microbiota in promoting rotavirus infection by depleting the mucus of IECs. Furthermore, metabolites from the gut microbiota may also play a crucial role in the defense against viruses. Emma et al. found that DCA, a secondary bile acid derived from the gut microbiota, could activate TLR7-MyD88 signaling and improve the production of type I interferon (IFN) through the IEC-plasmacytoid dendritic cell (pDC) axis, which restricts systemic Chikungunya virus (CHIKV) infection and potential transmission (Winkler et al., 2020). However, recent evidence indicated that SARS-CoV-2 infection could alter the composition of gut microbiota and SCFAs, but treatment with SCFAs did not change the replication or entry of SARS-CoV-2 in IECs, which suggested that microbiota-derived SCFAs do not interfere with SARS-CoV-2 infection in humans (Pascoal et al., 2021). In addition to viruses, signals from the gut microbiota to IECs also contribute to protection against parasite infection. For example, *Toxoplasma gondii* induces severe small intestinal inflammation, and the gut microbiota can trigger the autophagy of Paneth cells *via* induction of IFN-γ to maintain intestinal homeostasis during infection with *Toxoplasma gondii* (Burger et al., 2018). Other evidence suggests that the gut microbiota promotes the production of SPRR2A in Paneth cells and goblet cells *via* TLR-Myd88 signaling and protects the gut barrier during helminth infection (Hu et al., 2021).

In summary, gut microbiota or derived metabolites can influence IECs by signaling pathways during infection by bacteria, viruses, and parasites. However, the underlying mechanism is not yet clear, and more studies are required to elucidate the signals from the gut microbiota to IECs during infection.

### Regulation of the Gut Microbiota by IECs

Bacterial, viral and parasite infections can induce injury to the intestinal barrier through mucus degradation and disruption of tight junctions (Martens et al., 2018). In contrast, IECs directly resist pathogenic infection by secreting AMP, mucus, and hormones to form physical and biochemical barriers. However, the mechanisms of how IECs regulate gut microbiota and protect against infection are still unknown. Some studies suggested that IECs could influence the gut microbiota by altering the environment or signaling molecules during infection. For instance, *C. rodentium* injects type III secretion system effectors into IECs to target inflammation and establish infection, and IECs respond by rapidly shifting bioenergetic status to aerobic glycolysis, which induces a decline in obligate anaerobes and expansion of commensal *Enterobacteriaceae* (Mullineaux-Sanders et al., 2019). In addition, IECs can secrete fucosylated proteins into the lumen, and the gut microbiota use fucose as a nutrient, which improves host tolerance to *C. rodentium* infection (Pickard et al., 2014). Furthermore, infection can upregulate the expression of indoleamine 2,3-dioxygenase 1 (IDO1) in IECs, promote mucus production in goblet cells and increase the proportions of *Akkermansia muciniphila* and *Mucispirillum schaedleri via* AhR and Notch signals (Alvarado et al., 2019). Compared to normal mice, transgenic mice overexpressing IDO1 exhibited reduced infectious ileitis induced by *Escherichia coli*. Wang et al. demonstrated that the RNA helicase DEAD-box helicase 15 (Dhx15) mediates Wnt-induced antimicrobial protein expression in Paneth cells and that mice with IEC-specific depletion of Dhx15 are more susceptible to *Citrobacter rodentium* due to dysbiosis of the gut microbiota (Wang et al., 2021). However, IECs can also aggravate dysbiosis of the gut microbiota during inflammation. Rachael et al. found that IEC-derived reactive oxygen species (ROS) support the aerobic respiration of *E. coli* and enhance *E. coli* growth during gut inflammation, which indicated the double-sided effects of IECs on the gut microbiota during infections (Chanin et al., 2020). On the other hand, SARS-CoV-2 infects human cells through the angiotensin converting enzyme 2 (ACE2) receptor, and it has been suggested that ACE2 in IECs regulates gut microbiota composition and function (Viana et al., 2020). These results indicate that IECs can alter the gut microbiota through changes in the microenvironment and receptor modulation during infections, which indicates the crucial role of IECs in infection.

Taken together, viral, bacterial, and parasitic infections disturb the homeostasis of gut microbiota, alter the microenvironment of IECs and destroy the intestinal barrier. On the other hand, gut microbiota and IECs can directly and separately inhibit the invasion of pathogens through interactions with pathogens. In addition, the crosstalk between the gut microbiota and IECs also plays an essential role in combatting infections. However, some mechanisms of this crosstalk are still not clear. Understanding the crosstalk between IECs and the gut microbiota during infections may contribute to providing a novel strategy for prevention or treatment in patients with different infectious diseases, such as COVID-19.

## Underlying Prevention and Therapeutic Strategies

### Probiotics

Probiotics refer to live organisms that benefit human health. Emerging evidence has demonstrated that probiotics could improve the symptoms of some infectious diseases, such as CDI, infectious diarrhea, and necrotizing enterocolitis (McFarland, 2006; Alfaleh et al., 2010; Chen et al., 2010). Probiotics can maintain the homeostasis of the GIT by promoting mucus secretion from goblet cells, producing antibacterial factors and modulating the function of the gut barrier (Sherman et al., 2009). Many infectious diseases can induce dysbiosis of the gut microbiota and injury of the gut barrier, and treatment with probiotics may contribute to protecting the gut barrier from infection. For instance, *Escherichia coli* (*E. coli*) K1 causes gut barrier dysfunction by promoting apoptosis of IECs, reducing the expression of tight junction proteins, and increasing gut permeability. A probiotic mixture (containing *Bifidobacterium*, *Lactobacillus bulgaricus*, and *Streptococcus thermophilus*) can increase the production of mucin and decrease intestinal permeability to protect against *E. coli* K1 translocation (Zeng et al., 2017). In addition, *Enterococcus faecalis* (*E. faecalis*) is the most important nosocomial pathogen that causes systemic infection. Pipat et al. indicated that *E. faecalis* could translocate from the gut to the blood through production of the protease GelE, and oral administration of probiotic *Bacillus* spores blocked *E. faecalis* translocation by inhibiting the activity of fecal streptococci regulator (Fsr) (Piewngam et al., 2021). *C. difficile* infection poses an urgent threat to public health and causes more than 29,000 deaths in the United States every year (Chen et al., 2020). Chen et al. engineered the probiotic *Saccharomyces boulardii* to protect against primary and recurrent *C. difficile* infections, which may serve as a therapeutic for patients with infection of *C. difficile* (Chen et al., 2020). In addition to bacteria, probiotics also play a crucial role in viral infection. For example, a probiotic mixture (*Lactobacillus acidophilus* AD031 and *Bifidobacterium longum* BORI) can ameliorate the duration of fever and the frequency of diarrhea in infants with rotavirus infection (Park et al., 2017). In addition, administration with *Lactobacillus rhamnosus* improves diarrheal episodes in children with adenovirus infection (Freedman et al., 2021). Furthermore, substantial interest has emerged in novel strategies against COVID-19, such as probiotics. Although no published studies have demonstrated the efficacy of probiotics for COVID-19 treatment, some clinical trials using probiotics to improve the COVID-19 treatment efficacy are in progress (Lau et al., 2021). Collectively, probiotics can improve the dysbiosis of the gut microbiota and impairment of the gut barrier in different infections.

### Fecal Microbiota Transplantation

FMT is a method in which stool from healthy donors is placed into another patient’s intestine to normalize the microbiota composition, which brings a therapeutic benefit to recipients (Gupta and Khanna, 2017). Since 2013, the United States Food and Drug Administration has approved FMT for treating *Clostridium difficile* infections (Gupta and Khanna, 2017). Substantial interest has emerged in developing FMT as a therapeutic approach for metabolic syndrome, neurological diseases, and malignancies (Hanssen et al., 2021). For instance, Tanya et al. suggested that FMT ameliorates recurrent *Clostridioides difficile* infection by upregulating the expression of microRNAs in IECs (Monaghan et al., 2021). Cheng et al. demonstrated that FMT regulates the autophagy of IECs, modulates the composition of the gut microbiota and alleviates intestinal barrier injury in a piglet model with *Escherichia coli* K88 infection (Cheng et al., 2018). In addition, FMT also contributes to the treatment of virus infection. For example, alterations in the gut microbiota are linked to persistent inflammation during HIV infection, and FMT can increase the alpha diversity of the gut microbiota and attenuate HIV-associated dysbiosis in patients with HIV (Serrano-Villar et al., 2021). In addition, a recent study indicated the safety of FMT for recurrent *C. difficile* infection in patients with COVID-19 and speculated that intervention of gut microbiota *via* FMT may provide a novel strategy for COVID-19 (Biliński et al., 2021). However, further investigations are required to demonstrate the safety and efficacy of FMT, and more efforts should be undertaken to further standardize the FMT procedure.

### Dietary Fiber

**Figure 3:**
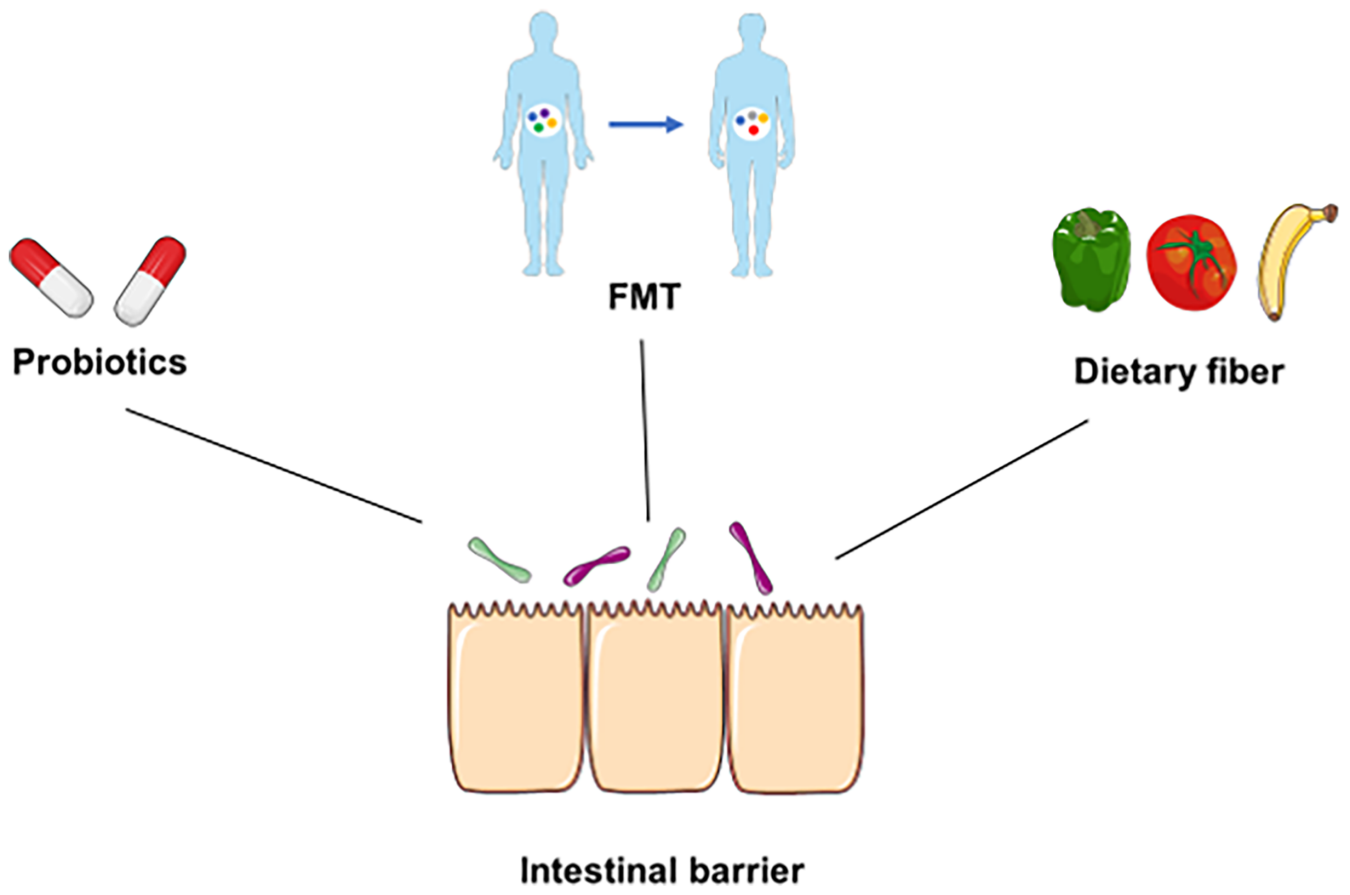
Underlying prevention and therapeutic strategies for infection. Some underlying prevention or therapeutic strategies, such as probiotics, FMT, and dietary fiber, can directly suppress pathogens, modulate the diversity of the gut microbiota, and maintain the integrity of the intestinal barrier during infection.

Dietary fiber is present in unrefined whole foods, such as vegetables, legumes, and fruits, and is not able to be digested by human alimentary enzymes (Holscher, 2017). Instead, dietary fiber can be metabolized by gut microbiota and generate SCFAs, such as propionate, acetate, and butyrate (Holscher, 2017). In addition, international guidelines suggest that dietary fiber may play an important role in the treatment of irritable bowel syndrome (IBS), diverticular disease, and IBD (Gill et al., 2021). On the other hand, emerging evidence indicates that dietary fiber can protect the GIT from pathogen infection. For instance, dietary fiber may directly inhibit enteric pathogens or indirectly influence infections by modulating the gut microbiota and immune function (Cao et al., 2021). Andrew et al. indicated that microbiota-accessible carbohydrates (MACs) decrease the expression of *C. difficile* toxin and suppress the infection of *C. difficile* in mice (Hryckowian et al., 2018). Furthermore, a recent study suggested that higher intakes of dietary fiber were associated with a lower susceptibility to SARS-CoV-2 infection, which indicated the prevention effect of dietary fiber in some infectious diseases, such as COVID-19 (Deschasaux-Tanguy et al., 2021). However, deprivation of dietary fiber induces dysbiosis of the gut microbiota, degrades the intestinal mucus barrier and enhances pathogen susceptibility (Desai et al., 2016). Deprivation of dietary fiber in specific pathogen-free mice promotes susceptibility to *C. rodentium* by altering the gut microbiota and disrupting mucosal barrier integrity (Neumann et al., 2021). Collectively, elucidating the unique repercussions of different interventions on enteric infections may help provide guidelines to prevent or alleviate enteric infection (**Figure 3**).

## Conclusions

In summary, the crosstalk between the gut microbiota plays an essential role in the maintenance of gut barrier function and homeostasis of the GIT under physiological conditions. Gut microbiota and derived metabolites compromise intestinal epithelium integrity through some signaling pathways, such as AhR, STAT3, and mTOR. In contrast, IECs form a physical and biochemical barrier to separate the gut microbiota from the host and regulate the gut microbiota by releasing mucins, AMPs, and hormones. However, different infections can induce dysbiosis of the gut microbiota and destroy the intestinal barrier. In that case, the interaction between gut microbiota and IECs contributes to the defense against pathogens. Some novel strategies, including probiotics, FMT, and dietary fiber, may provide underlying preventative or therapeutic effects for infectious diseases. Further studies should be conducted to discover the underlying mechanisms in the crosstalk between the gut microbiota and IECs during infection and to provide more therapeutic targets for infectious diseases.

## Author Contributions

All authors listed have made a substantial, direct, and intellectual contribution to the work, and approved it for publication.

## Funding

The study was supported by the Key projects of National Natural Science Foundation of China (No. 82030020), the National Natural Science Foundation of China (No. 81972327), and the National Natural Science Foundation of China (No. 81802477).

## Conflict of Interest

The authors declare that the research was conducted in the absence of any commercial or financial relationships that could be construed as a potential conflict of interest.

## Publisher’s Note

All claims expressed in this article are solely those of the authors and do not necessarily represent those of their affiliated organizations, or those of the publisher, the editors and the reviewers. Any product that may be evaluated in this article, or claim that may be made by its manufacturer, is not guaranteed or endorsed by the publisher.

## References

[B32] Alemi F. Poole D. P. Chiu J. Schoonjans K. Cattaruzza F. Grider J. R.. (2013). The Receptor TGR5 Mediates the Prokinetic Actions of Intestinal Bile Acids and Is Required for Normal Defecation in Mice. Gastroenterology 144, 145–154. doi: 10.1053/j.gastro.2012.09.055 23041323 PMC6054127

[B87] Alfaleh K. Anabrees J. Bassler D. (2010). Probiotics Reduce the Risk of Necrotizing Enterocolitis in Preterm Infants: A Meta-Analysis. Neonatology 97, 93–99. doi: 10.1159/000235684 19707025

[B10] Allam-Ndoul B. Castonguay-Paradis S. Veilleux A. (2020). Gut Microbiota and Intestinal Trans-Epithelial Permeability. Int. J. Mol. Sci. 21 (17), 6402. doi: 10.3390/ijms21176402 PMC7503654 32899147

[B42] Alvarado D. M. Chen B. Iticovici M. Thaker A. I. Dai N. VanDussen K. L.. (2019). Epithelial Indoleamine 2,3-Dioxygenase 1 Modulates Aryl Hydrocarbon Receptor and Notch Signaling to Increase Differentiation of Secretory Cells and Alter Mucus-Associated Microbiota. Gastroenterology 157, 1093–1108.e11. doi: 10.1053/j.gastro.2019.07.013 31325428 PMC6756966

[B58] Atarashi K. Tanoue T. Ando M. Kamada N. Nagano Y. Narushima S.. (2015). Th17 Cell Induction by Adhesion of Microbes to Intestinal Epithelial Cells. Cell 163, 367–380. doi: 10.1016/j.cell.2015.08.058 26411289 PMC4765954

[B39] Ayabe T. Satchell D. P. Wilson C. L. Parks W. C. Selsted M. E. Ouellette A. J. (2000). Secretion of Microbicidal Alpha-Defensins by Intestinal Paneth Cells in Response to Bacteria. Nat. Immunol. 1, 113–118. doi: 10.1038/77783 11248802

[B9] Berg D. Clemente J. C. Colombel J. F. (2015). Can Inflammatory Bowel Disease Be Permanently Treated With Short-Term Interventions on the Microbiome? Expert Rev. Gastroenterol. Hepatol. 9 (6), 781–795. doi: 10.1586/17474124.2015.1013031 25665875

[B100] Biliński J. Winter K. Jasiński M. Szczęś A. Bilinska N. Mullish B. H.. (2021). Rapid Resolution of COVID-19 After Faecal Microbiota Transplantation. Gut 71 (1), 230–232. doi: 10.1136/gutjnl-2021-325010 34230217

[B15] Bilotta A. J. Ma C. Yang W. Yu Y. Yu Y. Zhao X.. (2021). Propionate Enhances Cell Speed and Persistence to Promote Intestinal Epithelial Turnover and Repair. Cell Mol. Gastroenterol. Hepatol. 11, 1023–1044. doi: 10.1016/j.jcmgh.2020.11.011 33238220 PMC7898181

[B12] Blaak E. E. Canfora E. E. Theis S. Frost G. Groen A. K. Mithieux G.. (2020). Short Chain Fatty Acids in Human Gut and Metabolic Health. Benef. Microbes 11, 411–455. doi: 10.3920/BM2020.0057 32865024

[B78] Burger E. Araujo A. López-Yglesias A. Rajala M. W. Geng L. Levine B.. (2018). Loss of Paneth Cell Autophagy Causes Acute Susceptibility to Toxoplasma Gondii-Mediated Inflammation. Cell Host Microbe 23, 177–190.e4. doi: 10.1016/j.chom.2018.01.001 29358083 PMC6179445

[B103] Cao Y. Liu J. Zhu W. Qin N. Ren X. Zhu B.. (2021). Impact of Dietary Components on Enteric Infectious Disease. Crit. Rev. Food Sci. Nutr. 18, 1–26. doi: 10.1080/10408398.2021.1871587 33455435

[B83] Chanin R. B. Winter M. G. Spiga L. Hughes E. R. Zhu W. Taylor S. J.. (2020). Epithelial-Derived Reactive Oxygen Species Enable AppBCX-Mediated Aerobic Respiration of Escherichia Coli During Intestinal Inflammation. Cell Host Microbe 28, 780–788.e5. doi: 10.1016/j.chom.2020.09.005 33053375 PMC7736183

[B98] Cheng S. Ma X. Geng S. Jiang X. Li Y. Hu L.. (2018). Fecal Microbiota Transplantation Beneficially Regulates Intestinal Mucosal Autophagy and Alleviates Gut Barrier Injury. mSystems 3 (5), e00137–18. doi: 10.1128/mSystems.00137-18 PMC6178585 30320222

[B86] Chen C. C. Kong M. S. Lai M. W. Chao H. C. Chang K. W. Chen S. Y.. (2010). Probiotics Have Clinical, Microbiologic, and Immunologic Efficacy in Acute Infectious Diarrhea. Pediatr. Infect. Dis. J. 29, 135–138. doi: 10.1097/INF.0b013e3181b530bf 20135748

[B44] Chen Z. Luo J. Li J. Kim G. Chen E. S. Xiao S.. (2021). Foxo1 Controls Gut Homeostasis and Commensalism by Regulating Mucus Secretion. J. Exp. Med. 218 (9), e20210324. doi: 10.1084/jem.20210324 34287641 PMC8424467

[B91] Chen K. Zhu Y. Zhang Y. Hamza T. Yu H. Saint Fleur A.. (2020). A Probiotic Yeast-Based Immunotherapy Against Clostridioides Difficile Infection. Sci. Transl. Med. 12 (567), eaax4905. doi: 10.1126/scitranslmed.aax4905 33115949 PMC7692727

[B26] Chiang J. Y. (2009). Bile Acids: Regulation of Synthesis. J. Lipid Res. 50, 1955–1966. doi: 10.1194/jlr.R900010-JLR200 19346330 PMC2739756

[B57] Chung H. Pamp S. J. Hill J. A. Surana N. K. Edelman S. M. Troy E. B.. (2012). Gut Immune Maturation Depends on Colonization With a Host-Specific Microbiota. Cell 149, 1578–1593. doi: 10.1016/j.cell.2012.04.037 22726443 PMC3442780

[B63] Desai M. S. Seekatz A. M. Koropatkin N. M. Kamada N. Hickey C. A. Wolter M.. (2016). A Dietary Fiber-Deprived Gut Microbiota Degrades the Colonic Mucus Barrier and Enhances Pathogen Susceptibility. Cell 167, 1339–1353.e21. doi: 10.1016/j.cell.2016.10.043 27863247 PMC5131798

[B105] Deschasaux-Tanguy M. Srour B. Bourhis L. Arnault N. Druesne-Pecollo N. Esseddik Y.. (2021). Nutritional Risk Factors for SARS-CoV-2 Infection: A Prospective Study Within the NutriNet-Santé Cohort. BMC Med. 19, 290. doi: 10.1186/s12916-021-02168-1 34844606 PMC8629697

[B49] Donaldson G. P. Chou W. C. Manson A. L. Rogov P. Abeel T. Bochicchio J.. (2020). Spatially Distinct Physiology of Bacteroides Fragilis Within the Proximal Colon of Gnotobiotic Mice. Nat. Microbiol. 5, 746–756. doi: 10.1038/s41564-020-0683-3 32152589 PMC7426998

[B54] Ducarmon Q. R. Zwittink R. D. Hornung B. V. H. van Schaik W. Young V. B. Kuijper E. J. (2019). Gut Microbiota and Colonization Resistance Against Bacterial Enteric Infection. Microbiol. Mol. Biol. Rev. 83 (3), e00007–19. doi: 10.1128/MMBR.00007-19 PMC6710460 31167904

[B75] Engevik M. A. Banks L. D. Engevik K. A. Chang-Graham A. L. Perry J. L. Hutchinson D. S.. (2020). Rotavirus Infection Induces Glycan Availability to Promote Ileum-Specific Changes in the Microbiome Aiding Rotavirus Virulence. Gut Microbes 11, 1324–1347. doi: 10.1080/19490976.2020.1754714 32404017 PMC7524290

[B68] Fachi J. L. Felipe J. S. Pral L. P. da Silva B. K. Corrêa R. O. de Andrade M. C. P.. (2019). Butyrate Protects Mice From Clostridium Difficile-Induced Colitis Through an HIF-1-Dependent Mechanism. Cell Rep. 27, 750–761.e7. doi: 10.1016/j.celrep.2019.03.054 30995474

[B1] Fan Y. Pedersen O. (2021). Gut Microbiota in Human Metabolic Health and Disease. Nat. Rev. Microbiol. 19, 55–71. doi: 10.1038/s41579-020-0433-9 32887946

[B46] Flint H. J. Scott K. P. Duncan S. H. Louis P. Forano E. (2012). Microbial Degradation of Complex Carbohydrates in the Gut. Gut Microbes 3, 289–306. doi: 10.4161/gmic.19897 22572875 PMC3463488

[B93] Freedman S. B. Finkelstein Y. Pang X. L. Chui L. Tarr P. I. VanBuren J. M.. (2021). Pathogen-Specific Effects of Probiotics in Children With Acute Gastroenteritis Seeking Emergency Care: A Randomized Trial. Clin. Infect. Dis, 1:ciab876. doi: 10.1093/cid/ciab876 PMC9402642 34596225

[B38] Gehart H. Clevers H. (2019). Tales From the Crypt: New Insights Into Intestinal Stem Cells. Nat. Rev. Gastroenterol. Hepatol. 16, 19–34. doi: 10.1038/s41575-018-0081-y 30429586

[B102] Gill S. K. Rossi M. Bajka B. Whelan K. (2021). Dietary Fibre in Gastrointestinal Health and Disease. Nat. Rev. Gastroenterol. Hepatol. 18, 101–116. doi: 10.1038/s41575-020-00375-4 33208922

[B31] Golden J. M. Escobar O. H. Nguyen M. V. L. Mallicote M. U. Kavarian P. Frey M. R.. (2018). Ursodeoxycholic Acid Protects Against Intestinal Barrier Breakdown by Promoting Enterocyte Migration *via* EGFR- and COX-2-Dependent Mechanisms. Am. J. Physiol. Gastrointest. Liver Physiol. 315, G259–g271. doi: 10.1152/ajpgi.00354.2017 29672156 PMC6139640

[B55] Goto Y. Ivanov I. I. (2013). Intestinal Epithelial Cells as Mediators of the Commensal-Host Immune Crosstalk. Immunol. Cell Biol. 91, 204–214. doi: 10.1038/icb.2012.80 23318659 PMC3969236

[B59] Goto Y. Panea C. Nakato G. Cebula A. Lee C. Diez M. G.. (2014). Segmented Filamentous Bacteria Antigens Presented by Intestinal Dendritic Cells Drive Mucosal Th17 Cell Differentiation. Immunity 40, 594–607. doi: 10.1016/j.immuni.2014.03.005 24684957 PMC4084624

[B6] Greathouse K. L. White J. R. Vargas A. J. Bliskovsky V. V. Beck J. A. von Muhlinen N.. (2018). Interaction Between the Microbiome and TP53 in Human Lung Cancer. Genome Biol. 19, 123. doi: 10.1186/s13059-018-1501-6 30143034 PMC6109311

[B61] Grizotte-Lake M. Zhong G. Duncan K. Kirkwood J. Iyer N. Smolenski I.. (2018). Commensals Suppress Intestinal Epithelial Cell Retinoic Acid Synthesis to Regulate Interleukin-22 Activity and Prevent Microbial Dysbiosis. Immunity 49, 1103–1115.e6. doi: 10.1016/j.immuni.2018.11.018 30566883 PMC6319961

[B95] Gupta A. Khanna S. (2017). Fecal Microbiota Transplantation. Jama 318, 102. doi: 10.1001/jama.2017.6466 28672320

[B28] Halpern M. D. Holubec H. Saunders T. A. Dvorak K. Clark J. A. Doelle S. M.. (2006). Bile Acids Induce Ileal Damage During Experimental Necrotizing Enterocolitis. Gastroenterology 130, 359–372. doi: 10.1053/j.gastro.2005.10.023 16472592 PMC3417808

[B2] Han X. Lee A. Huang S. Gao J. Spence J. R. Owyang C. (2019). Lactobacillus Rhamnosus GG Prevents Epithelial Barrier Dysfunction Induced by Interferon-Gamma and Fecal Supernatants From Irritable Bowel Syndrome Patients in Human Intestinal Enteroids and Colonoids. Gut Microbes 10, 59–76. doi: 10.1080/19490976.2018.1479625 30040527 PMC6363076

[B96] Hanssen N. M. J. de Vos W. M. Nieuwdorp M. (2021). Fecal Microbiota Transplantation in Human Metabolic Diseases: From a Murky Past to a Bright Future? Cell Metab. 33, 1098–1110. doi: 10.1016/j.cmet.2021.05.005 34077717

[B5] Heijink I. H. Brandenburg S. M. Postma D. S. van Oosterhout A. J. (2012). Cigarette Smoke Impairs Airway Epithelial Barrier Function and Cell-Cell Contact Recovery. Eur. Respir. J. 39, 419–428. doi: 10.1183/09031936.00193810 21778164

[B20] Hinrichsen F. Hamm J. Westermann M. Schröder L. Shima K. Mishra N.. (2021). Microbial Regulation of Hexokinase 2 Links Mitochondrial Metabolism and Cell Death in Colitis. Cell Metab 33 (12), 2355–2366.e8. doi: 10.1016/j.cmet.2021.11.004 34847376

[B67] Hirota S. A. Fines K. Ng J. Traboulsi D. Lee J. Ihara E.. (2010). Hypoxia-Inducible Factor Signaling Provides Protection in Clostridium Difficile-Induced Intestinal Injury. Gastroenterology 139, 259–69.e3. doi: 10.1053/j.gastro.2010.03.045 20347817 PMC3063899

[B27] Hofmann A. F. (1999). The Continuing Importance of Bile Acids in Liver and Intestinal Disease. Arch. Intern. Med. 159, 2647–2658. doi: 10.1001/archinte.159.22.2647 10597755

[B101] Holscher H. D. (2017). Dietary Fiber and Prebiotics and the Gastrointestinal Microbiota. Gut Microbes 8, 172–184. doi: 10.1080/19490976.2017.1290756 28165863 PMC5390821

[B104] Hryckowian A. J. Van Treuren W. Smits S. A. Davis N. M. Gardner J. O. Bouley D. M.. (2018). Microbiota-Accessible Carbohydrates Suppress Clostridium Difficile Infection in a Murine Model. Nat. Microbiol. 3, 662–669. doi: 10.1038/s41564-018-0150-6 29686297 PMC6126909

[B21] Hubbard T. D. Murray I. A. Perdew G. H. (2015). Indole and Tryptophan Metabolism: Endogenous and Dietary Routes to Ah Receptor Activation. Drug Metab. Dispos. 43, 1522–1535. doi: 10.1124/dmd.115.064246 26041783 PMC4576673

[B56] Hu Z. Zhang C. Sifuentes-Dominguez L. Zarek C. M. Propheter D. C. Kuang Z.. (2021). Small Proline-Rich Protein 2A Is a Gut Bactericidal Protein Deployed During Helminth Infection. Science 374, eabe6723. doi: 10.1126/science.abe6723 34735226 PMC8977106

[B34] Ivanov I. I. Atarashi K. Manel N. Brodie E. L. Shima T. Karaoz U.. (2009). Induction of Intestinal Th17 Cells by Segmented Filamentous Bacteria. Cell 139, 485–498. doi: 10.1016/j.cell.2009.09.033 19836068 PMC2796826

[B18] Jin U. H. Cheng Y. Park H. Davidson L. A. Callaway E. S. Chapkin R. S.. (2017). Short Chain Fatty Acids Enhance Aryl Hydrocarbon (Ah) Responsiveness in Mouse Colonocytes and Caco-2 Human Colon Cancer Cells. Sci. Rep. 7, 10163. doi: 10.1038/s41598-017-10824-x 28860561 PMC5579248

[B33] Kayama H. Okumura R. Takeda K. (2020). Interaction Between the Microbiota, Epithelia, and Immune Cells in the Intestine. Annu. Rev. Immunol. 38, 23–48. doi: 10.1146/annurev-immunol-070119-115104 32340570

[B41] Kwon Y. H. Wang H. Denou E. Ghia J. E. Rossi L. Fontes M. E.. (2019). Modulation of Gut Microbiota Composition by Serotonin Signaling Influences Intestinal Immune Response and Susceptibility to Colitis. Cell Mol. Gastroenterol. Hepatol. 7, 709–728. doi: 10.1016/j.jcmgh.2019.01.004 30716420 PMC6462823

[B94] Lau H. C. Ng S. C. Yu J. (2021). Targeting the Gut Microbiota in Coronavirus Disease 2019: Hype or Hope? Gastroenterology 162 (1), 9–16. doi: 10.1053/j.gastro.2021.09.009 34508775 PMC8425294

[B70] Lei S. Samuel H. Twitchell E. Bui T. Ramesh A. Wen K.. (2016). Enterobacter Cloacae Inhibits Human Norovirus Infectivity in Gnotobiotic Pigs. Sci. Rep. 6, 25017. doi: 10.1038/srep25017 27113278 PMC4845002

[B35] Liang S. C. Tan X. Y. Luxenberg D. P. Karim R. Dunussi-Joannopoulos K. Collins M.. (2006). Interleukin (IL)-22 and IL-17 Are Coexpressed by Th17 Cells and Cooperatively Enhance Expression of Antimicrobial Peptides. J. Exp. Med. 203, 2271–2279. doi: 10.1084/jem.20061308 16982811 PMC2118116

[B71] Li D. Breiman A. le Pendu J. Uyttendaele M. (2015). Binding to Histo-Blood Group Antigen-Expressing Bacteria Protects Human Norovirus From Acute Heat Stress. Front. Microbiol. 6, 659. doi: 10.3389/fmicb.2015.00659 26191052 PMC4486850

[B47] Li H. Limenitakis J. P. Fuhrer T. Geuking M. B. Lawson M. A. Wyss M.. (2015). The Outer Mucus Layer Hosts a Distinct Intestinal Microbial Niche. Nat. Commun. 6, 8292. doi: 10.1038/ncomms9292 26392213 PMC4595636

[B16] Liu Z. Li L. Chen W. Wang Q. Xiao W. Ma Y.. (2018). Aryl Hydrocarbon Receptor Activation Maintained the Intestinal Epithelial Barrier Function Through Notch1 Dependent Signaling Pathway. Int. J. Mol. Med. 41 (3), 1560–1572. doi: 10.3892/ijmm.2017.3341 29286081 PMC5819918

[B43] Liu X. Lu J. Liu Z. Zhao J. Sun H. Wu N.. (2018). Intestinal Epithelial Cell-Derived LKB1 Suppresses Colitogenic Microbiota. J. Immunol. 200, 1889–1900. doi: 10.4049/jimmunol.1700547 29352002 PMC6814173

[B22] Li J. Zhang L. Wu T. Li Y. Zhou X. Ruan Z. (2021). Indole-3-Propionic Acid Improved the Intestinal Barrier by Enhancing Epithelial Barrier and Mucus Barrier. J. Agric. Food Chem. 69, 1487–1495. doi: 10.1021/acs.jafc.0c05205 33356219

[B8] Lynch S. V. Pedersen O. (2016). The Human Intestinal Microbiome in Health and Disease. N. Engl. J. Med. 375, 2369–2379. doi: 10.1056/NEJMra1600266 27974040

[B64] Mao T. Su C. W. Ji Q. Chen C. Y. Wang R. Vijaya Kumar D.. (2021). Hyaluronan-Induced Alterations of the Gut Microbiome Protects Mice Against Citrobacter Rodentium Infection and Intestinal Inflammation. Gut Microbes 13, 1972757. doi: 10.1080/19490976.2021.1972757 34592891 PMC8489935

[B19] Marinelli L. Martin-Gallausiaux C. Bourhis J. M. Béguet-Crespel F. Blottière H. M. Lapaque N. (2019). Identification of the Novel Role of Butyrate as AhR Ligand in Human Intestinal Epithelial Cells. Sci. Rep. 9, 643. doi: 10.1038/s41598-018-37019-2 30679727 PMC6345974

[B79] Martens E. C. Neumann M. Desai M. S. (2018). Interactions of Commensal and Pathogenic Microorganisms With the Intestinal Mucosal Barrier. Nat. Rev. Microbiol. 16, 457–470. doi: 10.1038/s41579-018-0036-x 29904082

[B13] Martin-Gallausiaux C. Marinelli L. Blottière H. M. Larraufie P. Lapaque N. (2021). SCFA: Mechanisms and Functional Importance in the Gut. Proc. Nutr. Soc. 80, 37–49. doi: 10.1017/S0029665120006916 32238208

[B85] McFarland L. V. (2006). Meta-Analysis of Probiotics for the Prevention of Antibiotic Associated Diarrhea and the Treatment of Clostridium Difficile Disease. Am. J. Gastroenterol. 101, 812–822. doi: 10.1111/j.1572-0241.2006.00465.x 16635227

[B97] Monaghan T. M. Seekatz A. M. Markham N. O. Yau T. O. Hatziapostolou M. Jilani T.. (2021). Fecal Microbiota Transplantation for Recurrent Clostridioides Difficile Infection Associates With Functional Alterations in Circulating microRNAs. Gastroenterology 161, 255–270.e4. doi: 10.1053/j.gastro.2021.03.050 33844988 PMC8579492

[B29] Mroz M. S. Lajczak N. K. Goggins B. J. Keely S. Keely S. J. (2018). The Bile Acids, Deoxycholic Acid and Ursodeoxycholic Acid, Regulate Colonic Epithelial Wound Healing. Am. J. Physiol. Gastrointest. Liver Physiol. 314, G378–g387. doi: 10.1152/ajpgi.00435.2016 29351391

[B11] Mukherji A. Kobiita A. Ye T. Chambon P. (2013). Homeostasis in Intestinal Epithelium Is Orchestrated by the Circadian Clock and Microbiota Cues Transduced by TLRs. Cell 153, 812–827. doi: 10.1016/j.cell.2013.04.020 23663780

[B80] Mullineaux-Sanders C. Sanchez-Garrido J. Hopkins E. G. D. Shenoy A. R. Barry R. Frankel G. (2019). Citrobacter Rodentium-Host-Microbiota Interactions: Immunity, Bioenergetics and Metabolism. Nat. Rev. Microbiol. 17, 701–715. doi: 10.1038/s41579-019-0252-z 31541196

[B106] Neumann M. Steimle A. Grant E. T. Wolter M. Parrish A. Willieme S.. (2021). Deprivation of Dietary Fiber in Specific-Pathogen-Free Mice Promotes Susceptibility to the Intestinal Mucosal Pathogen Citrobacter Rodentium. Gut Microbes 13, 1966263. doi: 10.1080/19490976.2021.1966263 34530674 PMC8451455

[B30] Pai R. Tarnawski A. S. Tran T. (2004). Deoxycholic Acid Activates Beta-Catenin Signaling Pathway and Increases Colon Cell Cancer Growth and Invasiveness. Mol. Biol. Cell 15, 2156–2163. doi: 10.1091/mbc.e03-12-0894 15004225 PMC404012

[B92] Park M. S. Kwon B. Ku S. Ji G. E. (2017). The Efficacy of Bifidobacterium Longum BORI and Lactobacillus Acidophilus AD031 Probiotic Treatment in Infants With Rotavirus Infection. Nutrients 9 (8), 887. doi: 10.3390/nu9080887 PMC5579680 28813007

[B77] Pascoal L. B. Rodrigues P. B. Genaro L. M. Gomes A. Toledo-Teixeira D. A. Parise P. L.. (2021). Microbiota-Derived Short-Chain Fatty Acids Do Not Interfere With SARS-CoV-2 Infection of Human Colonic Samples. Gut Microbes 13, 1–9. doi: 10.1080/19490976.2021.1874740 PMC7889267 33550892

[B74] Penninger J. M. Grant M. B. Sung J. J. Y. (2021). The Role of Angiotensin Converting Enzyme 2 in Modulating Gut Microbiota, Intestinal Inflammation, and Coronavirus Infection. Gastroenterology 160, 39–46. doi: 10.1053/j.gastro.2020.07.067 33130103 PMC7836226

[B52] Petri W. A. Jr. Miller M. Binder H. J. Levine M. M. Dillingham R. Guerrant R. L. (2008). Enteric Infections, Diarrhea, and Their Impact on Function and Development. J. Clin. Invest. 118, 1277–1290. doi: 10.1172/JCI34005 18382740 PMC2276781

[B81] Pickard J. M. Maurice C. F. Kinnebrew M. A. Abt M. C. Schenten D. Golovkina T. V.. (2014). Rapid Fucosylation of Intestinal Epithelium Sustains Host-Commensal Symbiosis in Sickness. Nature 514, 638–641. doi: 10.1038/nature13823 25274297 PMC4214913

[B90] Piewngam P. Chiou J. Ling J. Liu R. Pupa P. Zheng Y.. (2021). Enterococcal Bacteremia in Mice Is Prevented by Oral Administration of Probiotic Bacillus Spores. Sci. Transl. Med. 13, eabf4692. doi: 10.1126/scitranslmed.abf4692 34818053 PMC11097119

[B65] Pral L. P. Fachi J. L. Corrêa R. O. Colonna M. Vinolo M. A. R. (2021). Hypoxia and HIF-1 as Key Regulators of Gut Microbiota and Host Interactions. Trends Immunol. 42, 604–621. doi: 10.1016/j.it.2021.05.004 34171295 PMC8283795

[B51] Qu D. Wang G. Yu L. Tian F. Chen W. Zhai Q. (2021). The Effects of Diet and Gut Microbiota on the Regulation of Intestinal Mucin Glycosylation. Carbohydr. Polym. 258, 117651. doi: 10.1016/j.carbpol.2021.117651 33593539

[B72] Ren Z. Wang H. Cui G. Lu H. Wang L. Luo H.. (2021). Alterations in the Human Oral and Gut Microbiomes and Lipidomics in COVID-19. Gut 70, 1253–1265. doi: 10.1136/gutjnl-2020-323826 33789966 PMC8042598

[B4] Samarkos M. Mastrogianni E. Kampouropoulou O. (2018). The Role of Gut Microbiota in Clostridium Difficile Infection. Eur. J. Intern. Med. 50, 28–32. doi: 10.1016/j.ejim.2018.02.006 29428498

[B23] Scott S. A. Fu J. Chang P. V. (2020). Microbial Tryptophan Metabolites Regulate Gut Barrier Function *via* the Aryl Hydrocarbon Receptor. Proc. Natl. Acad. Sci. U. S. A. 117, 19376–19387. doi: 10.1073/pnas.2000047117 32719140 PMC7431026

[B7] Sender R. Fuchs S. Milo R. (2016). Revised Estimates for the Number of Human and Bacteria Cells in the Body. PLoS Biol. 14, e1002533. doi: 10.1371/journal.pbio.1002533 27541692 PMC4991899

[B99] Serrano-Villar S. Talavera-Rodríguez A. Gosalbes M. J. Madrid N. Pérez-Molina J. A. Elliott R. J.. (2021). Fecal Microbiota Transplantation in HIV: A Pilot Placebo-Controlled Study. Nat. Commun. 12, 1139. doi: 10.1038/s41467-021-21472-1 33602945 PMC7892558

[B88] Sherman P. M. Ossa J. C. Johnson-Henry K. (2009). Unraveling Mechanisms of Action of Probiotics. Nutr. Clin. Pract. 24, 10–14. doi: 10.1177/0884533608329231 19244144

[B50] Shin J. Noh J. R. Chang D. H. Kim Y. H. Kim M. H. Lee E. S.. (2019). Elucidation of Akkermansia Muciniphila Probiotic Traits Driven by Mucin Depletion. Front. Microbiol. 10, 1137. doi: 10.3389/fmicb.2019.01137 31178843 PMC6538878

[B3] Soderholm A. T. Pedicord V. A. (2019). Intestinal Epithelial Cells: At the Interface of the Microbiota and Mucosal Immunity. Immunology 158, 267–280. doi: 10.1111/imm.13117 31509239 PMC6856932

[B62] Stacy A. Andrade-Oliveira V. McCulloch J. A. Hild B. Oh J. H. Perez-Chaparro P. J.. (2021). Infection Trains the Host for Microbiota-Enhanced Resistance to Pathogens. Cell 184, 615–627.e17. doi: 10.1016/j.cell.2020.12.011 33453153 PMC8786454

[B24] Swimm A. Giver C. R. DeFilipp Z. Rangaraju S. Sharma A. Ulezko Antonova A.. (2018). Indoles Derived From Intestinal Microbiota Act *via* Type I Interferon Signaling to Limit Graft-Versus-Host Disease. Blood 132, 2506–2519. doi: 10.1182/blood-2018-03-838193 30257880 PMC6284212

[B40] Vaishnava S. Yamamoto M. Severson K. M. Ruhn K. A. Yu X. Koren O.. (2011). The Antibacterial Lectin RegIIIgamma Promotes the Spatial Segregation of Microbiota and Host in the Intestine. Science 334, 255–258. doi: 10.1126/science.1209791 21998396 PMC3321924

[B25] Venkatesh M. Mukherjee S. Wang H. Li H. Sun K. Benechet A. P.. (2014). Symbiotic Bacterial Metabolites Regulate Gastrointestinal Barrier Function *via* the Xenobiotic Sensor PXR and Toll-Like Receptor 4. Immunity 41, 296–310. doi: 10.1016/j.immuni.2014.06.014 25065623 PMC4142105

[B84] Viana S. D. Nunes S. Reis F. (2020). ACE2 Imbalance as a Key Player for the Poor Outcomes in COVID-19 Patients With Age-Related Comorbidities - Role of Gut Microbiota Dysbiosis. Ageing Res. Rev. 62, 101123. doi: 10.1016/j.arr.2020.101123 32683039 PMC7365123

[B37] von Moltke J. Ji M. Liang H. E. Locksley R. M. (2016). Tuft-Cell-Derived IL-25 Regulates an Intestinal ILC2-Epithelial Response Circuit. Nature 529, 221–225. doi: 10.1038/nature16161 26675736 PMC4830391

[B82] Wang Y. He K. Sheng B. Lei X. Tao W. Zhu X.. (2021). The RNA Helicase Dhx15 Mediates Wnt-Induced Antimicrobial Protein Expression in Paneth Cells. Proc. Natl. Acad. Sci. U. S. A. 118 (4), e2017432118. doi: 10.1073/pnas.2017432118 33483420 PMC7848544

[B36] Wang Y. Kuang Z. Yu X. Ruhn K. A. Kubo M. Hooper L. V. (2017). The Intestinal Microbiota Regulates Body Composition Through NFIL3 and the Circadian Clock. Science 357, 912–916. doi: 10.1126/science.aan0677 28860383 PMC5702268

[B53] Wang C. Li Q. Ren J. (2019). Microbiota-Immune Interaction in the Pathogenesis of Gut-Derived Infection. Front. Immunol. 10, 1873. doi: 10.3389/fimmu.2019.01873 31456801 PMC6698791

[B76] Winkler E. S. Shrihari S. Hykes B. L. Jr. Handley S. A. Andhey P. S. Huang Y. S.. (2020). The Intestinal Microbiome Restricts Alphavirus Infection and Dissemination Through a Bile Acid-Type I IFN Signaling Axis. Cell 182, 901–918.e18. doi: 10.1016/j.cell.2020.06.029 32668198 PMC7483520

[B48] Wlodarska M. Luo C. Kolde R. d'Hennezel E. Annand J. W. Heim C. E.. (2017). Indoleacrylic Acid Produced by Commensal Peptostreptococcus Species Suppresses Inflammation. Cell Host Microbe 22, 25–37.e6. doi: 10.1016/j.chom.2017.06.007 28704649 PMC5672633

[B60] Woo V. Eshleman E. M. Hashimoto-Hill S. Whitt J. Wu S. E. Engleman L.. (2021). Commensal Segmented Filamentous Bacteria-Derived Retinoic Acid Primes Host Defense to Intestinal Infection. Cell Host Microbe 29 (12), 1744–1756.e5. doi: 10.1016/j.chom.2021.09.010 34678170 PMC8667605

[B45] Yang L. Liu C. Zhao W. He C. Ding J. Dai R.. (2018). Impaired Autophagy in Intestinal Epithelial Cells Alters Gut Microbiota and Host Immune Responses. Appl. Environ. Microbiol. 84 (18), e00880–18. doi: 10.1128/AEM.00880-18 PMC6121970 30006408

[B69] Yap Y. A. McLeod K. H. McKenzie C. I. Gavin P. G. Davalos-Salas M. Richards J. L.. (2021). An Acetate-Yielding Diet Imprints an Immune and Anti-Microbial Programme Against Enteric Infection. Clin. Transl. Immunol. 10, e1233. doi: 10.1002/cti2.1233 PMC7809703 33489123

[B73] Yeoh Y. K. Zuo T. Lui G. C. Zhang F. Liu Q. Li A. Y.. (2021). Gut Microbiota Composition Reflects Disease Severity and Dysfunctional Immune Responses in Patients With COVID-19. Gut 70, 698–706. doi: 10.1136/gutjnl-2020-323020 33431578 PMC7804842

[B66] Yin J. Zhou C. Yang K. Ren Y. Qiu Y. Xu P.. (2020). Mutual Regulation Between Butyrate and Hypoxia-Inducible Factor-1α in Epithelial Cell Promotes Expression of Tight Junction Proteins. Cell Biol. Int. 44, 1405–1414. doi: 10.1002/cbin.11336 32129567

[B17] Yu M. Wang Q. Ma Y. Li L. Yu K. Zhang Z.. (2018). Aryl Hydrocarbon Receptor Activation Modulates Intestinal Epithelial Barrier Function by Maintaining Tight Junction Integrity. Int. J. Biol. Sci. 14, 69–77. doi: 10.7150/ijbs.22259 29483826 PMC5821050

[B89] Zeng Q. He X. Puthiyakunnon S. Xiao H. Gong Z. Boddu S.. (2017). Probiotic Mixture Golden Bifido Prevents Neonatal Escherichia Coli K1 Translocation *via* Enhancing Intestinal Defense. Front. Microbiol. 8, 1798. doi: 10.3389/fmicb.2017.01798 28979247 PMC5611410

[B14] Zhao Y. Chen F. Wu W. Sun M. Bilotta A. J. Yao S.. (2018). GPR43 Mediates Microbiota Metabolite SCFA Regulation of Antimicrobial Peptide Expression in Intestinal Epithelial Cells *via* Activation of mTOR and STAT3. Mucosal Immunol. 11, 752–762. doi: 10.1038/mi.2017.118 29411774 PMC5976519

